# Prevalence and correlates of proteinuria in Kampala, Uganda: a cross-sectional pilot study

**DOI:** 10.1186/s13104-016-1897-6

**Published:** 2016-02-16

**Authors:** Joseph Lunyera, John W. Stanifer, Prossie Ingabire, Wilson Etolu, Peace Bagasha, Joseph R. Egger, Uptal D. Patel, Gerald Mutungi, Robert Kalyesubula

**Affiliations:** Duke Global Health Institute, Duke University, 310 Trent Drive, Durham, NC USA; School of Medicine, Makerere University College of Health Sciences, Kampala, Uganda; Department of Medicine, Duke Clinical Research Institute and Duke Global Health Institute, Duke University, Durham, NC USA; Department of Medicine, Mulago Hospital, Makerere University College of Health Sciences, Kampala, Uganda; Departments of Medicine and Pediatrics, Duke Clinical Research Institute and Duke Global Health Institute, Duke University, Durham, NC USA; Section of Non-communicable Diseases, Ministry of Health, Kampala, Uganda; Department of Physiology, Makerere University College of Health Sciences, Kampala, Uganda

**Keywords:** Proteinuria, Kidney disease, Epidemiology, Uganda, Non-communicable diseases, Sub-Saharan Africa

## Abstract

**Background:**

Despite the increasing prevalence of chronic kidney disease (CKD) in sub-Saharan Africa, few community-based screenings have been conducted in Uganda. Opportunities to improve the management of CKD in sub-Saharan Africa are limited by low awareness, inadequate access, poor recognition, and delayed presentation for clinical care. Therefore, the Uganda Kidney Foundation engaged key stakeholders in performing a screening event on World Kidney Day.

**Methods:**

We conducted a cross-sectional pilot study in March 2013 from a convenience sample of adult, urban residents in Kampala, Uganda. We advertised the event using radio and television announcements, newspapers, billboards, and notice boards at public places, such as places of worship. Subsequently, we screened for proteinuria, hypertension, fasting glucose impairment, and obesity in a central and easily-accessible location.

**Results:**

We enrolled 141 adults most of whom were female (57 %), young (64 %; 18–39 years), and had a professional occupation (52 %). The prevalence of proteinuria (13 %; 95 % confidence interval [CI] 7–19 %), hypertension (38 %; 95 % CI 31–47 %), and impaired fasting glucose (13 %; 95 % CI 9–20 %) were high in this study population. Proteinuria was most prevalent among young (18–39 years) adults (n = 14; 16 %) and among those who reported a history of alcohol intake (n = 10; 32 %).

**Conclusions:**

The prevalence of proteinuria was high among a convenience sample of urban residents in a sub-Saharan African setting. These results represent an important effort by the Ugandan Kidney Foundation to increase awareness and recognition of CKD, and they will help formulate additional epidemiological studies on NCDs in Uganda which are urgently needed and now feasible.

## Background

Chronic kidney disease (CKD) is a growing health burden globally, with many known and unknown etiologies [1]. In sub-Saharan Africa (SSA), the epidemiology of CKD has not been well described [2], and in Uganda, few community-based studies assessing kidney function have been conducted. Nonetheless, recent epidemiological studies in SSA suggest that the prevalence is substantial, with estimates as high as 24 % in Zambia and 15 % in some urban areas of Tanzania [2–7].

Opportunities to improve the management of CKD in SSA are limited by low awareness, inadequate access, poor recognition, and delayed presentation for clinical care [8]. As such, the morbidity and mortality from CKD are significant especially considering the lack of cardiovascular care and inadequate access to renal replacement therapies (RRT), such as peritoneal dialysis, hemodialysis, or kidney transplantation [9]. In Uganda, hemodialysis services are only available in limited urban centers (0.1 dialysis machine/100,000 population) and there are no active kidney transplant programs [9]. In 2011, kidney disease was the 13th most common cause of death in Uganda, with the estimated annual age-adjusted death rate attributable to CKD in Uganda (27.8 per 100,000 population) [10] exceeding the global estimate (16.3 per 100,000) [1].

Given the limited understanding of kidney disease in Uganda, the Uganda Kidney Foundation was founded on March 13th 2013 to advocate, increase awareness, and improve care for kidney disease in Uganda. To achieve this mission, demonstrating the feasibility of screening studies that can describe the epidemiology of kidney disease are urgently needed. Therefore, as part of the International Society of Nephrology World Kidney Day Campaign on March 13th 2013 in Kampala, we performed a community-based pilot study to raise awareness by assessing kidney function through proteinuria measurements.

## Methods

### Ethical approval

Ethical approval for this study was granted by the Mulago Hospital Ethical Review Board. Informed consent was obtained from all participants prior to their enrollment.

### Study design, participants, and site

We conducted a cross-sectional pilot study in March 2013 from a convenience sample of adult (≥18 years old) urban residents in Kampala, Uganda. Kampala is the largest city in Uganda with a population of more than 1.5 million people [11]. Most residents belong to the Bantu ethnic group, but Nilotes and other ethnicities are also common [12].

Prior to the study, we advertised using radio and television announcements, newspapers, billboards, and noticeboards at public places, such as places of worship. The advertisements were designed to produce a study catchment area that included all the five divisions of Kampala [11]. To be eligible for the study, individuals had to be overnight fasting and report no prior medical history of kidney disease. We enrolled consecutive individuals who met these inclusion criteria. The study site was located on the campus of Makerere University College of Health Sciences (MakCHS), School of Medicine, which is located 3 km from the city center and is easily accessible.

### Data collection

We collected socio-demographic characteristics from interviewer-administered surveys, as well as anthropometric and blood pressure measurements, random blood glucose (RBG) using a finger-prick glucose assay, and a mid-stream clean-catch urine for urine protein. Individuals who screened positive for disease were provided appropriate referral to Mulago Hospital for further clinical care.

For the anthropometric measurements, weight was measured in kilograms (kg) using a pre-calibrated weighing scale (Seca scale). Height was measured in centimeters (cm) using a standardized stadiometer. Body mass index (BMI) was calculated from the weight and height measurements for respondents as follows: BMI = (weight/height^2^). Overweight was defined as BMI between 25 and 30 kg/m^2^; obesity was defined as BMI ≥30 kg/m^2^.

Blood pressure was measured using automated sphygmomanometers that were calibrated according to the manufacturer’s recommendations. Blood pressure was measured after 10 min rest in a seated position. A second blood pressure measurement was taken at a minimum 5 min interval from the initial measurement. Systolic hypertension was defined as an average systolic blood pressure (SBP) ≥140 mmHg, and diastolic hypertension was defined as an average diastolic blood pressure (DBP) ≥90 mmHg. Hypertension was defined as the presence of systolic or diastolic hypertension, or on-going use of blood pressure lowering medicines among those with self-reported history of hypertension.

Finger-prick blood samples were obtained to measure random blood glucose using the Accu-Chek Active glucometer (Roche Diagnostics, Germany), which was calibrated according to the manufacturer’s recommendation. Fasting glucose impairment was defined as RBG >126 mg/dl, or on-going use of anti-diabetic medicines among those who reported having diabetes. Respondents also provided a clean-catch midstream urine specimen in sterile containers for a dipstick urinalysis (Combur Test M, Roche Diagnostics, Germany). Proteinuria was defined as a urine protein of ≥1+ on dipstick in the absence of hematuria and leukocyturia.

### Statistical analysis

Data were manually entered into a Microsoft Excel spreadsheet. All entries were double-checked for accuracy by two independent study personnel, and then exported to STATA v.13 software (Stata corp., College Station, TX) for analyses. Continuous variables were summarized by their median and inter-quartile range (IQR) and categorical variables as crude counts and percentages. We estimated the crude, unweighted prevalence of proteinuria and other CKD co-morbidities with 95 % confidence intervals (CI). We used generalized linear models with a log link to report prevalence ratios (PR) and explore associations between proteinuria, hypertension, obesity, impaired fasting glucose, and socio demographic and lifestyle characteristics. A p value of <0.05 was considered statistically significant.

A secondary interest of the study was to identify high risk groups in the community. We estimated the potential benefit of screening among these high risk groups as the number needed to screen (NNS) in order to identify one case of proteinuria, which was defined as the reciprocal of the absolute risk reduction [13]. Using Bender’s method, we constructed a 95 % CI around the NNS estimates [14]. Confidence intervals were truncated at zero.

## Results

### Characteristics of study participants

We enrolled 141 adults, most of whom were female (n = 81; 57 %), 18–39 years of age (n = 87; 64 %), and had a professional occupation (n = 71; 52 %). Fewer than half reported a history of alcohol intake (n = 54; 39 %), and only two (1 %) reported a history of smoking. Few respondents had a self-reported medical history of hypertension (n = 21; 15 %), diabetes (n = 8; 6 %), or heart disease (n = 4; 3 %) (Table 1). These demographics were similar to the population of Kampala where most people are female (53 %) and 18–39 years of age (61 %), and where many people are occupied as wage earners (24 %), with few being unemployed (4 %) [11, 15].

**Table 1:** Characteristics of participants by proteinuria status (n = 141), Kampala, Uganda; 2013

Demographic and clinical characteristics	All, n = 141	With proteinuria, n = 18 (13 %)	Without proteinuria, n = 121 (87 %)	Crude PR (95 % CI)	Adjusted PR^a^ (95 % CI)
Age					
18–39 years old	87	14 (16 %)	72 (84 %)	3.74 (0.89–15.77)	7.96 (1.39–45.44)
40–59 years old	46	2 (4 %)	44 (96 %)	Ref	Ref
≥60 years old	4	0	4 (100 %)	–	–
Gender					
Male	60	9 (15 %)	50 (85 %)	1.36 (0.57–3.21)	–
Female	81	9 (11 %)	71 (89 %)	Ref	–
Occupation					
Unemployed	6	1 (17 %)	5 (83 %)	Ref	–
Student	27	2 (7 %)	25 (93 %)	–	–
Wage earner^b^	33	7 (21 %)	26 (79 %)	1.35 (0.30–6.10)	–
Professional^c^	71	7 (10 %)	63 (90 %)	2.25 (0.24–20.96)	–
History of Smoking	2	0	2 (100 %)	–	–
History of alcohol intake	54	11 (20 %)	43 (80 %)	2.44 (1.01–5.92)	3.92 (1.10–14.03)
18–39 years old	31	10 (32 %)	21 (68 %)	4.35 (1.49–12.7)	–
40–59 years old	20	1 (5 %)	19 (95 %)	1.30 (0.09–19.5)	–
>60 years old	2	0	2 (100 %)	–	–
Self-reported medical history					
Hypertension	21	1 (5 %)	20 (95 %)	–	–
Diabetes mellitus	8	2 (25 %)	6 (75 %)	–	–
Heart disease^d^	4	0	4 (100 %)	–	–
Body mass index					
Normal weight	58	8 (14 %)	50 (86 %)	Ref	–
Overweight	81	10 (12 %)	71 (88 %)	1.24 (0.49–3.14)	–
Obese	40	3 (8 %)	37 (92 %)	0.54 (0.15–1.93)	–
Hypertension	54	9 (17 %)	45 (83 %)	1.57 (0.67–3.71)	1.50 (0.41–5.83)
Systolic hypertension	38	8 (21 %)	30 (79 %)	2.13 (0.91–4.98)	–
Diastolic hypertension	29	5 (17 %)	24 (83 %)	1.46 (0.57–3.76)	–
Impaired fasting glucose	19	5 (26 %)	14 (74 %)	2.43 (0.98–6.04)	1.64 (0.28–9.73)

*PR* prevalence ratio

^a^Co-variates: gender, occupation, hypertension, impaired fasting glucose

^b^Wage earner: any non-salaried position (e.g., construction worker, farmer)

^c^Professional: any salaried position (e.g. nurse, teacher, government employee)

^d^Heart disease: coronary disease, heart failure, or structural diseases

The median BMI of respondents was 25.9 kg/m^2^ (IQR 22.7–30.7). More than half of respondents were overweight (n = 83; 59 %; 95 % CI 50–67 %) and many (n = 42; 30 %; 95 % CI 22–37 %) were obese. The median SBP was 128 (IQR 120–138) mmHg, and median DBP was 78 (IQR 71–85) mmHg. The prevalence of hypertension was 38 % (95 % CI 31–47 %). Among participants with hypertension (n = 54), systolic hypertension was more prevalent (27 %; 95 % CI 20–34 %) than diastolic hypertension (21 %; 95 % CI 14–27 %). The median random blood glucose for participants was 91 (IQR 80–105) mg/dl, and 19 (13 %; 95 % CI 9–20 %) participants had impaired fasting glucose. Eighteen participants (13 %; 95 % CI 7–19 %) had proteinuria, of whom 16 had proteinuria of 1+ (89 %) and 2 (11 %) had proteinuria ≥2+ **(**Table 1).

The majority of participants diagnosed with medical conditions during the screening were not aware of having these conditions. Of the 43 participants newly diagnosed with hypertension at the screening, most (n = 32; 74 %) were unaware of having hypertension, and of the 10 participants with impaired fasting glucose, most (n = 7; 70 %) were unaware.

### Epidemiology of proteinuria, hypertension, and impaired fasting glucose

Proteinuria was most prevalent among young (18–39 years) adults (n = 14; 16 %), and it was more prominent in those who reported a history of alcohol intake (n = 10; 32 %). It was also most prevalent among adults with impaired fasting glucose (n = 3; 30 %), those who reported a history of diabetes (n = 2; 25 %), those with systolic hypertension (n = 8; 22 %), and those occupied as wage earner (n = 7; 21 %) (Table 1). There was no significant difference in the prevalence of proteinuria by gender. Proteinuria was less prevalent among participants with self reported medical history of hypertension (n = 1; 5 %), among older adults aged 40–59 years old (n = 2; 4 %) or ≥60 years old (n = 0), and among adults with self-reported history of smoking (n = 0) or heart disease (n = 0).

In univariable regression, self-reported history of alcohol intake (PR = 2.44; 1.01–5.92) and younger age (18–39 years old) (PR = 3.74, 0.89–15.77) were significantly associated with proteinuria. There was no significant association between proteinuria and impaired fasting glucose, hypertension, overweight, obesity, smoking, gender, or occupation (Table 1). When stratified by age, the significant association between alcohol intake and proteinuria was most prominent among younger (18–39 years) adults (PR = 4.35, 1.49–12.70). In multi-variable regression, the association between alcohol intake and proteinuria (PR = 3.92; 95 % CI 1.10–14.03) persisted despite adjustment for occupation, hypertension, and impaired fasting glucose.

### Potential benefits of targeted screening for proteinuria

The number needed to screen in order to identify one case of proteinuria in this convenience sample of urban residents in Kampala was eight among young (18–39 years old) adults, six among young (18–39 years old) adults who were occupied as professionals, and four among young (18–39 years old) adults who had hypertension. Among adults who report a history of alcohol intake, the number needed to screen to identify one case of proteinuria was eight across all age groups, and four among young (18–39 years) adults.

## Discussion

Our pilot study is one of the first to assess kidney function, fasting glucose impairment, hypertension, and obesity in a community setting in Uganda. The prevalence of proteinuria was high in this study population, and it was most prevalent among younger participants. Concurrent with this high prevalence was low disease awareness for hypertension and impaired fasting glucose.

Although non-communicable diseases (NCDs) such as diabetes and hypertension account for most cases of CKD in high-income countries [1], many recent studies in low- and middle-income countries (LMICs) have shown that CKD may only be partly attributable to these NCDs [6]. Other potential etiologies for CKD in LMICs include herbal medicines, environmental toxins, such as heavy metals and agrochemicals, as well as many other unmeasured factors [1, 6, 7, 16]. Our findings are consistent with these observations, and they highlight a need to explore potential etiologies of CKD, e.g., the role that alcohol or other environmental toxins may play in the development of proteinuria among young adults.

In LMICs, CKD affects younger portions of the population which reflects the different population demographics observed in these countries [1, 7, 8]. Our study, which found a significantly higher prevalence among younger age groups, highlights the urgent need for epidemiologic studies capable of identifying high-risk populations. Proteinuria is an important predictor of risk of progression to end-stage renal disease as well as cardiovascular disease [17–19]. As such, understanding its epidemiology, especially if it disproportionately affects younger, economically productive adults, is critically important in developing public health agendas in Uganda. Moreover, because of the limited availability and readiness of the health system in Uganda to care for chronic diseases such as CKD [20], screening for proteinuria, which is an efficient, reliable, and easily obtainable means of assessing kidney function, may be an effective strategy, particularly in lower level health facilities where other kidney function measurements are not available [21–23].

In response to the need for increasing awareness and screening for NCDs such as CKD, the Uganda Kidney Foundation has used various platforms and strategies to engage key stakeholders, such as physicians, nurses, medical officers, and public health officials in the Ministry of Health. The Uganda Kidney Foundation also partnered with professional societies including the International Society of Nephrology (ISN) to organize the first international kidney conference in Kampala in 2012 to increase awareness and care for kidney disease among clinicians [24]. Because CKD often goes undetected, collaborative efforts to increase awareness and to screen at-risk community members may be the most effective and cost-efficient public health strategies to address the burden of CKD, particularly in a LMIC like Uganda [25–29].

Our study has many strengths. The screening activity was carried out by internal medicine residents at MakCHS, who obtained relevant medical history from respondents during the screening to exclude data from respondents with proteinuria secondary to other medical conditions, such as urinary tract infections (UTIs). Additionally, with limited resources, we were able to screen for proteinuria, hypertension, impaired fasting glucose, and obesity using standard definitions and diagnostic tests.

Despite these strengths, we noted several limitations. Because urine dipstick measurements for proteinuria can be non-specific, we could not exclude orthostatic or transient proteinuria, or evaluate for possible glomerulonephritis, and we did not repeat the proteinuria assessment. Additionally, we did not assess kidney function using serum creatinine and estimated glomerular filtration rate, and the cross-sectional design prevents any causal assumptions about etiologies. For example, the association between alcohol intake and proteinuria that we observed could be due to unmeasured confounding effects. Because we obtained a convenience sample of people who attended the World Kidney Day celebrations in Kampala, our prevalence estimates are not generalizable to the Kampala urban population. As such, our confidence intervals are intended to measure the variance of the observed sample, and not the underlying Kampala general population. Selection bias was also a concern. Health-conscious individuals were more likely to participate in screening activities than the general population. Nonetheless, our pilot findings highlight the urgent need for and feasibility of future epidemiological studies pertaining to CKD and other NCDs in Uganda.

## Conclusions

We observed a high prevalence of proteinuria in a convenience sample of urban residents in a sub-Saharan African setting. These results are among the first to be reported for a community-based sample in Uganda, and they represent an important effort by the Uganda Kidney Foundation to increase awareness and recognition of CKD. These estimates will help formulate additional epidemiological studies on NCDs in Uganda which are urgently needed.
